# Follicular development and endometrial receptivity of different androgen phenotypes in women with polycystic ovary syndrome

**DOI:** 10.3389/fendo.2024.1400880

**Published:** 2024-12-12

**Authors:** Xinling Wen, Li Wang, Shulan Lv

**Affiliations:** ^1^ Department of Anesthesiology and Operation, The First Affiliated Hospital of Xi’an Jiaotong University, Xi’an, Shaanxi, China; ^2^ Department of Gynecology and Obstetrics, The First Affiliated Hospital of Xi’an Jiaotong University, Xi’an, Shaanxi, China

**Keywords:** polycystic ovary syndrome, follicular development, endometrial receptivity, hyperandrogenism, reproductive-aged women, infertility

## Abstract

**Objective:**

Polycystic ovary syndrome (PCOS) is an important factor contributing to infertility in reproductive-aged women. Hyperandrogenism (HA) plays an important role in the pathogenesis of PCOS. This study was conducted to explore the follicular development and endometrial receptivity of different androgen phenotypes in reproductive-aged patients with PCOS.

**Methods:**

A total of 268 PCOS patients with infertility were recruited and divided into two groups according to the different androgen phenotypes in this study: abnormal menstruation and hyperandrogenism (AM-HA group, *n* = 127) and abnormal menstruation and polycystic ovarian morphology (AM-PCOM group, *n* = 141). The follicular development, endometrial receptivity, pregnancy rate, and live birth rate during the natural menstrual cycle were compared between the two groups.

**Results:**

The number of dominant follicles, number of ovulations, and normal ovulation rate in the AM-HA group were significantly lower compared with those in the AM-PCOM group (*p* < 0.05). The endometrial thickness (ET), endometrial volume (EV), vascularization index (VI), flow index (FI), and vascularization flow index (VFI) on days 14 to 24 of the menstrual cycle before ovulation were significantly lower in the AM-HA group than in the AM-PCOM group (*p* < 0.05). The endometrial VI, FI, and VFI, the integrin αvβ3, and VEGF concentrations in the uterine fluid during the implantation window were significantly lower in the AM-HA group compared with the AM-PCOM group (*p* < 0.05). However, no statistically significant differences were observed in the uterine artery blood flow parameters, ET and EV, between the two groups (*p* > 0.05). The biochemical pregnancy rate, clinical pregnancy rate, ongoing pregnancy rate, and live birth rate in the AM-HA group were significantly lower compared with those in the AM-PCOM group (*p* < 0.05).

**Conclusion:**

PCOS patients with the AM-HA phenotype were vulnerable to ovulation disorders and impaired endometrial receptivity, which resulted in reduced pregnancy rate. Treatment with HA is likely to become an effective approach for improving endometrial receptivity and fecundity disorders in patients with PCOS.

## Introduction

Polycystic ovary syndrome (PCOS) is a common reproductive endocrine and metabolic disorder in reproductive-aged women (1). Epidemiological investigations have reported that the incidence of PCOS is 5%–15% (2). There is a high degree of heterogeneity among patients with PCOS worldwide, including menstrual irregularities, hyperandrogenism (HA), anovulatory infertility, and metabolic disorders (1). The diagnostic criteria for PCOS have been proposed by different organizations over the past several decades, among which the Rotterdam criterion has been the most widely used diagnostic criterion (3). However, even when the same diagnostic criteria for PCOS are used, the incidence rate is significantly different due to various factors, including study population, race, living environment, dietary habits, and a lack of standardized definitions for the phenotypes (4). In addition, the clinical manifestations of HA differ greatly between European and Asian countries (5). The diagnostic criteria and guidelines for PCOS are constantly updated. Building on the 2018 international evidence-based guidelines for the assessment and management of PCOS, the recommendations from the 2023 international evidence-based guidelines for the assessment and management of PCOS updated and expanded clinical questions, which aimed to support women and their healthcare providers in optimizing the diagnosis, assessment, and management of PCOS (6).

PCOS is an important factor contributing to infertility in reproductive-aged women and accounts for 70%–80% of women with anovulatory infertility (7). In addition to ovulation dysfunction, reduced endometrial receptivity is also an important factor leading to fecundity disorders in PCOS patients (8). Thin endometrium, reduced blood supply to the endometrium, abnormal decidualization of endometrial stromal cells, aberrant expression of sex hormones and their receptors in the endometrium, and decreased molecular markers of endometrial receptivity all likely contribute to reduced endometrial receptivity and infertility (9). Human embryo implantation involves complex and multifactor interactions, and endometrial receptivity plays an important role in embryo implantation. Our previous studies confirmed that ultrasonic parameters and biomarkers in uterine secretions during the implantation window were non-invasive predictor markers for endometrial receptivity (10, 11).

The Androgen Excess Society (AES) proposed that HA was a necessary condition for the diagnosis of PCOS (12). Studies have confirmed that HA plays an important role in the pathogenesis of PCOS (13). Furthermore, HA and insulin resistance (IR) interact with each other to form a vicious cycle in PCOS. Different androgen phenotypes may have different effects on fertility, including follicle development, ovulation, and endometrial function. The epidemiological and diagnostic criteria of PCOS have been a concern in the field of gynecology endocrinology. This study primarily explored the mechanisms behind the effects of androgen phenotypes on fertility outcomes in reproductive-aged patients with PCOS, including follicular development and endometrial receptivity of natural menstrual cycle.

## Materials and methods

### Study design and participants

A total of 268 PCOS patients with infertility were recruited for this study at the First Affiliated Hospital of Xi’an Jiaotong University from February 2020 to June 2022. The diagnostic criteria for PCOS we used were the Rotterdam criteria: (a) chronic ovulatory dysfunction (OD), (b) clinical manifestations or biochemical evidence of HA, and (c) PCOM: the presence of at least 12 antral follicles measuring 2–9 mm in diameter in the unilateral ovary or bilateral ovaries, and (or) an increased ovarian volume (≥10 mL). PCOS could be diagnosed when any two of these three criteria were presented (14). HA was diagnosed according to either clinical manifestations or laboratory evidence. Hirsutism was defined as a modified Ferriman–Gallwey score of more than 3 on physical examination (6). Laboratory evidence was defined as an abnormally increased testosterone level (>1.67 nmol/L). All PCOS patients in this study had abnormal menstruation, including oligomenorrhea or secondary amenorrhea. Oligomenorrhea was identified as women with menstrual cycle >35 days (15). Secondary amenorrhea was considered as women with normal menstrual frequency who stopped menstruation for 3 months or those with oligomenorrhea who stopped menstruation for 6 months (16). Participants were excluded if they had thyroid disease, adrenal disease, autoimmune disease, or fallopian tube blocking, or if their husband had abnormal semen. In addition, participants were also excluded if they received treatment with hormone drugs in the past 6 months.

The baseline characteristics of patients were recorded, including age, blood pressure, body mass index (BMI), modified Ferriman–Gallwey score, waist circumference, hip circumference, waist-to-hip ratio (WHR), infertility duration, and family history. The basal concentrations of serum sex hormone on 2–4 days of the menstrual cycle, and anti- Müllerian hormone (AMH) concentration were detected in the clinical laboratory of our hospital using chemiluminescence method. Lifestyle guidance from a professional was given to all participants during the study, including diet and physical activity.

### Outcome measures

According to the different androgen phenotypes, all patients were divided into two groups: the abnormal menstruation and HA group (AM-HA group, *n* = 127) and the abnormal menstruation and PCOM group (AM-PCOM group, *n* = 141). Women in the AM-HA group did not have PCOM, and women in the AM-PCOM group did not have HA. The follicle growth and parameters of endometrial receptivity were monitored by transvaginal sonography during the natural menstrual cycle. The ultrasonic parameters included pulsatility index (PI) and resistance index (RI) of the uterine artery, endometrial thickness (ET), endometrial volume (EV), endometrial flow index (FI), endometrial vascularization index (VI), and endometrial vascularization flow index (VFI). The data of all participants were collected in a standardized manner at the same time of the menstrual cycle to ensure data consistency. For PCOS patients with oligomenorrhea, the data were collected from the 8th day of the menstrual cycle. For PCOS patients with secondary amenorrhea, the data were collected from the 8th day of progesterone withdrawal bleeding. When one dominant follicle reached 18 mm in diameter, human chorionic gonadotropin (hCG) at a dose of 10,000 IU was given intramuscularly to trigger ovulation and timed intercourse was advised. Normal ovulation was defined according to the following criteria (10): (a) the average diameter of dominant follicle reached at least 18 mm, and then the dominant follicle disappeared, (b) the midluteal serum progesterone was ≥15 nmol/L. Luteinized unruptured follicular syndrome (LUFS) was considered as persistent existence or expansion of mature follicle, and the follicle wall is thickened (17). The ultrasonic parameters of endometrial receptivity were examined in all participants from the 8th day to the 24th day of the menstrual cycle. When ovulation occurred or no dominant follicle was observed until the 24th day of the natural menstrual cycle, the ultrasound examinations were completed. All ultrasonic scans were performed by one operator to avoid inter-observer variation. The ultrasonic parameters were measured three times, and the average value was used for the final statistical analysis.

The ultrasonic parameters and biochemical parameters of endometrial receptivity during the implantation window of patients with normal ovulation between the two groups were detected. The uterine fluid was collected through the embryo transfer catheter, and then the samples were centrifuged and frozen at −20°C for the detection of biochemical indicators. The integrin αvβ3 and vascular endothelial growth factor (VEGF) concentrations in the uterine fluid were tested by enzyme-linked immunosorbent assay (ELISA) (18).

Exploring endometrial receptivity parameters is crucial to the success of pregnancy, especially during the implantation window. Therefore, the primary outcome was the endometrial receptivity during the embryo implantation window (defined as 6 to 7 days after ovulation) between PCOS patients with different androgen phenotypes. The secondary outcomes included follicular development, ovulation rate, endometrial receptivity before ovulation, pregnancy rates, and live birth rates.

Biochemical pregnancy was considered as the serum hCG level exceeded 10 mIU/mL 2 weeks after ovulation. Clinical pregnancy was identified as the appearance of pregnant bursa or embryo in the uterine cavity. Ongoing pregnancy was considered as the appearance of fetal cardiac activity at 12 weeks of pregnancy (19). Pregnancy rate = number of pregnancies/number of participants in each group × 100%. A live birth was defined as an infant born showing any signs of life, at least ≥ 20 weeks gestational age and weighing 500 g. Live birth rates = number of live births/number of participants in each group×100%. The pregnancy rates and live birth rates during the natural menstrual cycle were analyzed in this study.

### Statistical analysis

The data in this study were analyzed using SPSS version 20.0. The Kolmogorov–Smirnov test was used to check the normal distribution prior to statistical tests. For normally distributed variables, the continuous variables were given as mean ± standard deviation and analyzed by Student’s *t* test, whereas the Mann–Whitney *U* test was used to analyze non-normally distributed data. Categorical data were given as number and percentage (%), which were analyzed by the chi-square test. When the expected frequencies fell below five, the dichotomous data were analyzed by Fisher’s exact test. A value of *p* < 0.05 was considered statistically significant.

## Results

### Baseline characteristics of PCOS patients with different phenotypes

**Table 1** shows the baseline characteristics of patients between the two groups. The Ferriman–Gallwey score and serum testosterone concentration were significantly higher in the AM-HA group compared with the AM-PCOM group (*p* < 0.05). No statistically significant differences were observed in the other baseline characteristics between the two groups (*p* > 0.05).

**Table 1:** Baseline characteristics of different phenotypes in PCOS patients.

Parameters	AM-HA group (n = 127)	AM-PCOM group (n = 141)	*p-*value
Age (years)	33.6 ± 6.1	34.9 ± 6.3	0.803
Ferriman–Gallwey score	5.4 ± 1.9	2.5 ± 0.9	0.026
Blood pressure (mmHg)			
Systolic blood pressure	102.5 ± 11.7	104.6 ± 13.5	0.823
Diastolic blood pressure	71.6 ± 9.8	72.3 ± 8.9	0.901
BMI (kg/m^2^)	23.8 ± 6.5	22.7 ± 5.3	0.642
WHR	0.8 ± 0.2	0.8 ± 0.3	0.935
Infertility duration (years)	2.2 ± 0.9	2.0 ± 0.7	0.862
Types of abnormal menstruation			0.459
Oligomenorrhea	98 (77.2)	114 (80.9)	
Secondary amenorrhea	29 (22.8)	27 (19.1)	
Family history			
Diabetes mellitus	19 (15.0)	23 (16.3)	0.761
Hypertension	14 (11.0)	16 (11.3)	0.933
Coronary heart disease	11 (8.7)	10 (7.1)	0.633
Basal concentration			
FSH (mIU/mL)	6.5 ± 1.9	5.9 ± 2.1	0.714
LH (mIU/mL)	15.3 ± 3.7	13.1 ± 3.2	0.677
PRL (ng/mL)	12.6 ± 2.4	14.3 ± 3.1	0.590
E_2_ (pmol/L)	96.7 ± 15.8	85.5 ± 19.2	0.645
P (nmol/L)	1.1 ± 0.6	1.0 ± 0.7	0.992
T (nmol/L)	2.5 ± 0.9	1.5 ± 0.8	0.031
AMH (ng/mL)	4.2 ± 0.9	3.9 ± 0.8	0.807
Day of hCG administration	20.6 ± 3.7	20.2 ± 3.4	0.905

Data given as mean ± SD or number (%).

FSH, follicle-stimulating hormone; LH, luteinizing hormone; PRL, prolactin; P, progesterone; E_2_, estradiol; T, testosterone.

### Follicular development and ovulation rate

**Figure 1:**
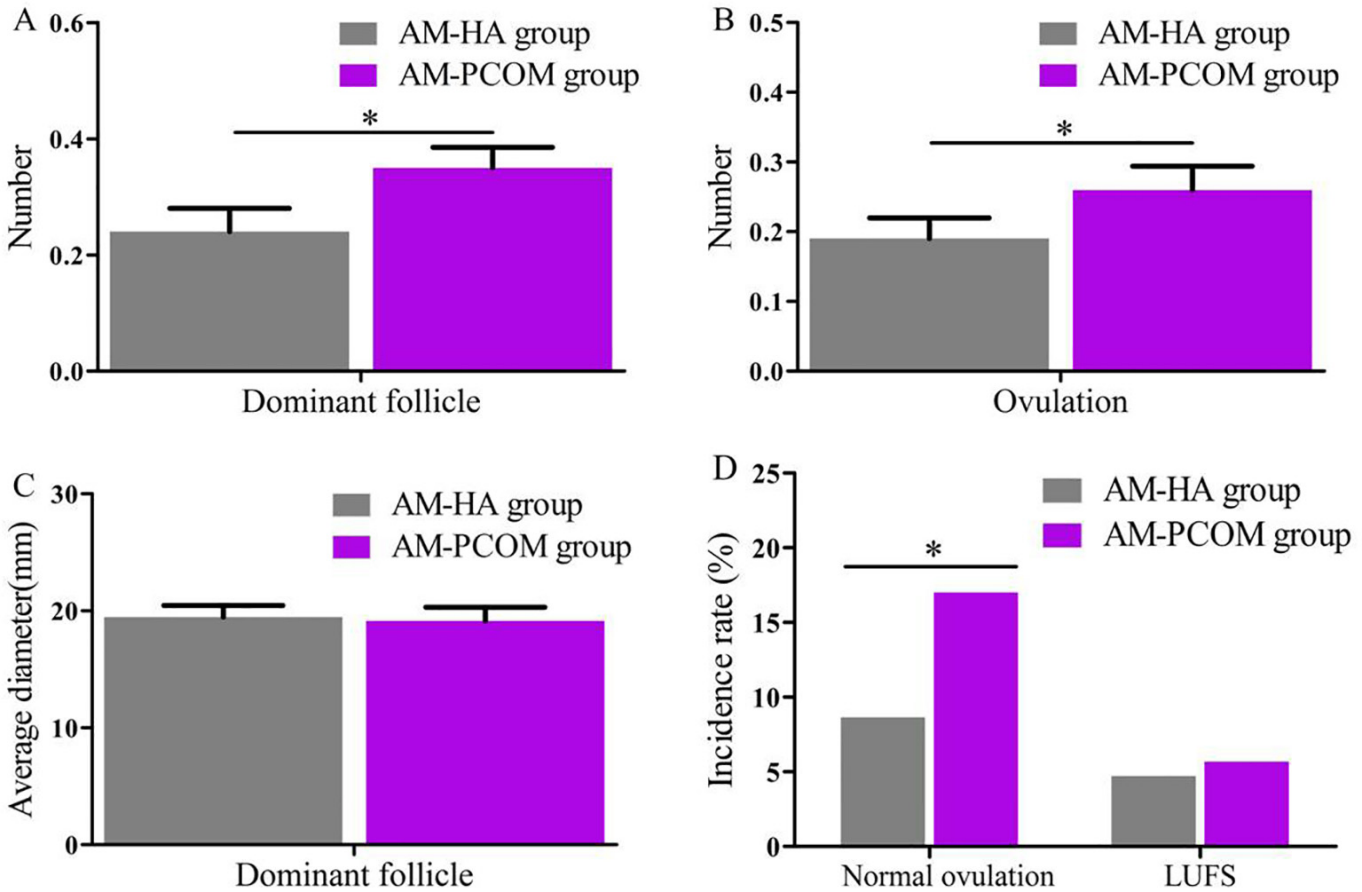
Comparison of follicular development and ovulation rate between the two groups. **(A)** Number of dominant follicle. **(B)** Number of ovulation. **(C)** Average diameter of dominant follicle. **(D)** Incidence rate of normal ovulation and LUFS (*p < 0.05).

The data in **Figure 1** illustrate that the number of dominant follicles, number of ovulations, and normal ovulation rate in the AM-HA group were significantly lower than those in the AM-PCOM group (*p* < 0.05). However, no statistically significant differences were observed in the average diameter of the dominant follicle and LUFS rate between the two groups (*p* > 0.05).

### Endometrial receptivity before ovulation

**Figure 2:**
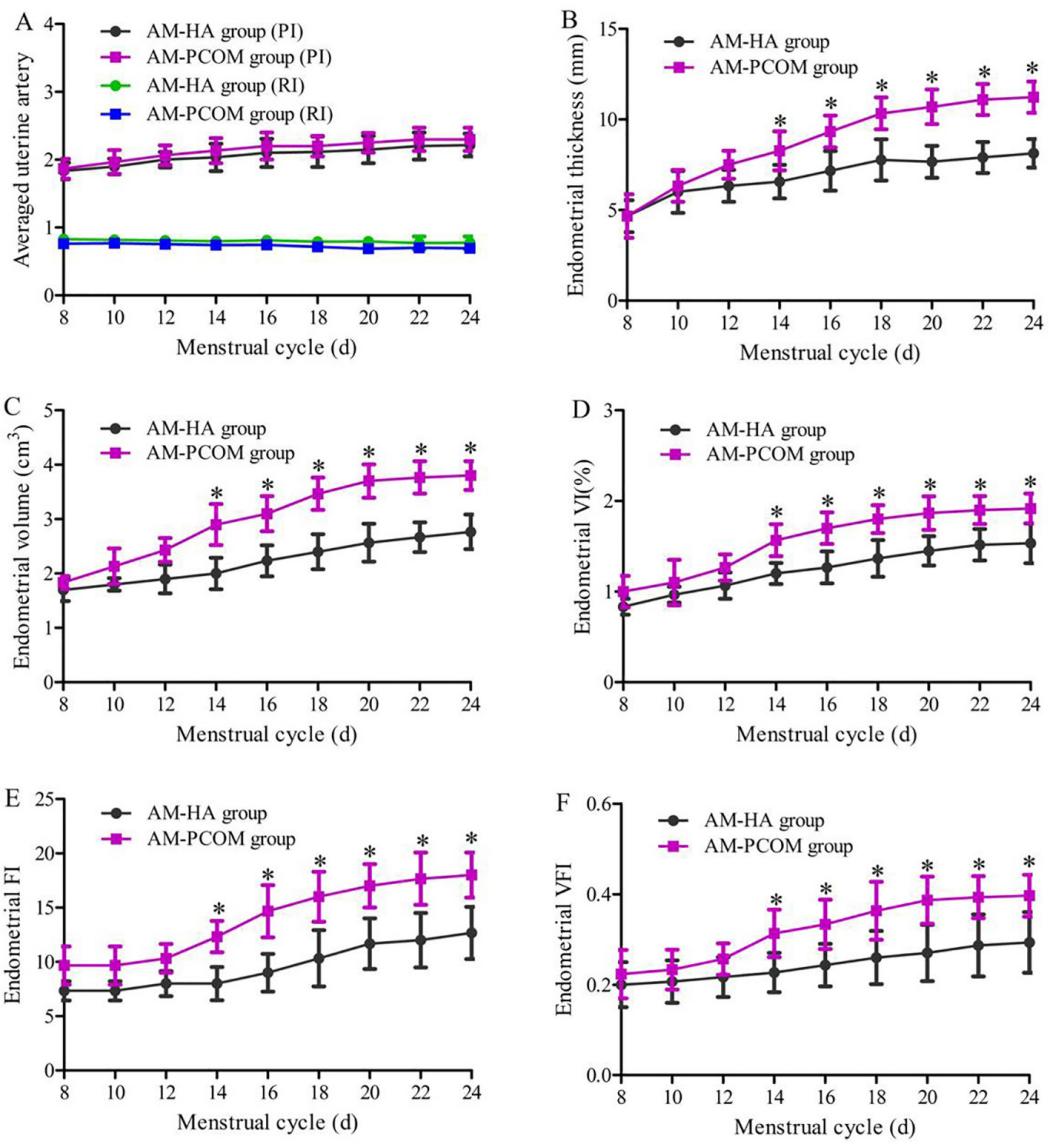
Ultrasonic parameters of endometrial receptivity before ovulation during the natural menstrual cycle between the two groups. **(A)** Uterine artery blood flow parameters. **(B)** Endometrial thickness. **(C)** Endometrial volume. **(D)** Endometrial VI. **(E)** Endometrial FI. **(F)** Endometrial VFI (*p < 0.05).

**Figure 2** demonstrates the ultrasonic parameters of endometrial receptivity before ovulation during the natural menstrual cycle between the two groups. Data show that the ET, EV, VI, FI, and VFI on days 14 to 24 of the menstrual cycle were significantly lower in the AM-HA group than in the AM-PCOM group (*p* < 0.05). However, no statistically significant differences were observed in the averaged uterine artery PI and RI between the two groups (*p* > 0.05).

### Endometrial receptivity during the implantation window

**Figure 3:**
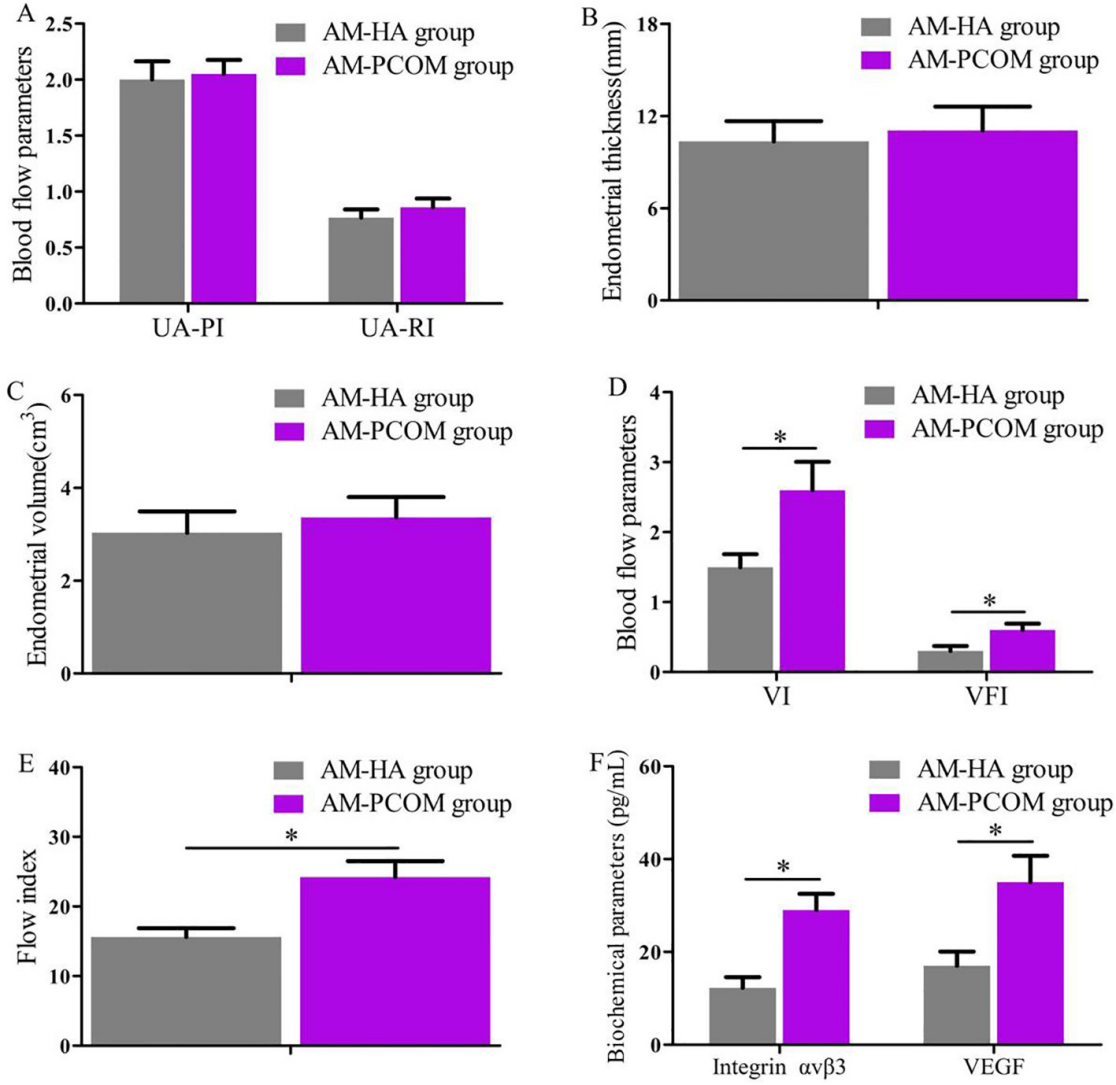
Comparison of endometrial receptivity parameters during the implantation window between the two groups. **(A)** Uterine artery blood flow parameters. **(B)** Endometrial thickness. **(C)** Endometrial volume. **(D)** Endometrial VI and VFI. **(E)** Endometrial FI. **(F)** Biochemical parameter concentrations in uterine fluid. UA-PI, uterine artery pulsatility index; UA-RI, uterine artery resistance index; VI, vascularization index; VFI, vascularization flow index (*p < 0.05).

The endometrial receptivity during the implantation window of patients with normal ovulation between the two groups was compared and displayed in **Figure 3**. The VI, FI, and VFI were significantly lower in the AM-HA group than those in the AM-PCOM group (*p* < 0.05), and the integrin αvβ3 and VEGF concentrations in the uterine fluid were significantly lower in the AM-HA group than those in the AM-PCOM group (*p* < 0.05). However, no statistically significant differences were observed in the uterine artery blood flow parameters, ET and EV, between the two groups (*p* > 0.05).

### Pregnancy rates

**Table 2** presents the pregnancy rates of different phenotypes in patients with PCOS. The biochemical pregnancy rate, clinical pregnancy rate, and ongoing pregnancy rate in the AM-HA group were significantly lower than those in the AM-PCOM group (*p* < 0.05).

**Table 2:** Pregnancy rates of different phenotypes in patients with PCOS.

Parameters	AM-HA group (n = 127)	AM-PCOM group (n = 141)	*p-*value
Biochemical pregnancy	5 (3.9)	15 (10.6)	0.037
Clinical pregnancy	4 (3.1)	14 (9.9)	0.027
Ongoing pregnancy	4 (3.1)	13 (9.2)	0.042

Data were given as number (%).

### Live birth rates

Four pregnant patients in the AM-HA group delivered healthy newborns at full term, with a live birth rate of 3.1% (4/127). One pregnant patient in the AM-PCOM group delivered prematurely at 35 weeks of gestation due to premature membrane rupture, and the other 12 pregnant patients delivered healthy newborns at full term. Therefore, the live birth rate of patients in the AM-PCOM group was 9.2% (13/141). The live birth rate of patients in the AM-HA group was significantly lower compared with patients in the AM-PCOM group (*p* < 0.05).

## Discussion

The primary sources of circulating androgens in female patients include direct ovarian production and the peripheral transition of adrenal sex steroid precursors to active androgens. Androgen plays an important role in female reproduction, in both healthy and pathological states. Androgens are regulated through the androgen receptor (AR) and are important for normal follicular development, endometrial receptivity, embryo implantation, and female fertility (20). AR is expressed during follicle development in oocytes, granulosa cells, theca cells, and stromal cells (21). Animal studies have shown that AR expression in granulosa cells is critical for normal follicular development and subsequent ovulation (22). Nevertheless, in addition to the positive effects of normal concentrations of androgen on follicular development, HA seriously inhibit normal follicular development and maturation. However, relevant data on follicle development and endometrial receptivity of different phenotypes in patients with PCOS are limited so far. The participants in this study were recruited from the same region, and with the same race and similar socioeconomic background, which ensured consistency in diagnostic criteria and baseline measurements. Women with PCOS in this study were divided into the AM-HA group and the AM-PCOM group. Follicular development, ovulation, and endometrial receptivity were compared between the two groups. The ultrasonic parameters of endometrial receptivity were detected not only by two-dimensional ultrasound but also through new three-dimensional energy Doppler ultrasound. In addition, biochemical indicators of endometrial receptivity during the implantation window in patients with normal ovulation between the two groups were detected. These methods are currently non-invasive methods for evaluating endometrial receptivity. The objective of this study was to observe the effects of HA and PCOM phenotypes on follicular development and endometrial receptivity in patients with PCOS, so as to provide basis for individualized clinical treatment plans.

Our findings indicated that the number of dominant follicles, number of ovulations, and normal ovulation rate in the AM-HA group were significantly lower than those in the AM-PCOM group. The mechanism of follicular development is complex, which is regulated by endocrine, paracrine, and appropriate intrafollicular microenvironments (1). FSH, AMH, estrogen, and androgen are essential for the growth and development of ovarian follicles. HA is one of the important features of PCOS, and its clinical manifestations include hirsutism, acne, and seborrheic alopecia (23). As a double-edged sword, testosterone plays dual roles in the regulation of follicular growth and development, depending on its concentration (24). Low concentrations of testosterone can promote the recruitment of follicles, facilitate follicles from the reserve pool to the growth and development pool, and combine with testosterone receptors to accelerate the proliferation of follicular stromal cells and granulosa cells. However, high concentrations of testosterone have opposite effects on follicular growth, including inhibiting the growth and development of sinus follicles, and inducing follicular apoptosis and atresia (25).

HA inhibits follicular development and ovulation in a variety of ways. First, testosterone receptors exist in the granuloidal cells of presinus follicles (26). Testosterone is the substrate of FSH-induced aromatization reaction. Low concentrations of testosterone promotes aromatase to produce estrogen, thus facilitating follicular development. However, when the testosterone concentrations are too high, the activity of aromatase can be inhibited, and the growth and development of follicles can subsequently be suppressed. Second, after the transformation of testosterone to dihydrotestosterone (DHT), which is strongly affected by 5α reductase, it inhibits the development and maturity of follicles (27). Androgens that over-recruited sinus follicles promote granulosa cells to secrete AMH and other follicle growth inhibitory factors through the interaction between follicles, reduce the sensitivity of follicles to FSH, and then prevent the continuous growth, development, and maturation of follicles (28). Third, the high androgen environment in the follicular fluid of PCOS patients inhibits the expression of aquaporin-9 (AQP9) in granulosa cells through the phosphatidylinositol 3 kinase pathway, thereby preventing follicular maturation and ovulation (29, 30). Menstrual dysfunction and HA are associated with each other. HA causes premature development of ovarian follicles, forms multiple small antral follicles in ovaries, then causes anovulation and infertility. Therefore, PCOS patients with HA should be treated before pregnancy to improve fertility.

Suitable endometrial receptivity is crucial for embryo adhesion and implantation, and impaired endometrial receptivity has been proven to be an important factor for infertility and spontaneous abortion. At present, endometrial receptivity is evaluated by various methods, including two-dimensional or three-dimensional ultrasound and biochemical indicators in uterine secretions and endometrial tissue. The most common of which is transvaginal ultrasound due to its being non-invasive. Uterine secretions contain various cytokines, which provide an appropriate microenvironment for embryo implantation (31). In addition, the safety of the uterine secretion collection method has been confirmed by our previous studies and other literature reports, among which the selected participants included patients with unexplained infertility, women who accepted intrauterine insemination or *in vitro* fertilization-embryo transfer (IVF-ET), infertile patients with endometriosis, and patients with idiopathic infertility or luteal phase deficiency (9, 10). Data in this study indicated that ET, EV, VI, FI, and VFI on days 14 to 24 before ovulation during the natural menstrual cycle were significantly lower in the AM-HA group than in the AM-PCOM group. In addition, data in our study showed that the endometrial VI, FI, and VFI, the integrin αvβ3, and VEGF concentrations in uterine fluid during the implantation window were significantly lower in the AM-HA group compared with the AM-PCOM group. These results suggested that the phenotype of AM-HA in PCOS patients was vulnerable to impaired endometrial receptivity, which resulted in reduced pregnancy rate. Hence, the treatment for HA is likely to become an effective approach for improving endometrial receptivity and fecundity disorders in patients with PCOS.

This study also compared the pregnancy rates between PCOS patients with different androgen phenotypes. Our findings displayed that the biochemical pregnancy rate, clinical pregnancy rate, and ongoing pregnancy rate in the AM-HA group were significantly lower than in the AM-PCOM group. Furthermore, the live birth rate of patients in the AM-HA group was significantly lower than that of patients in the AM-PCOM group. Studies have shown that estrogen receptor-alpha (ER-α) expression level in the endometrium is significantly higher in patients with unexplained infertility compared to the control women (32). In addition, previous studies have reported that the expression level of ER-α is negatively correlated with the expression level of HOXA10 in the endometrium, and that increased expression of ER-α inhibits the decidualization of mouse endometrium during early pregnancy (33). The results of the PCOS animal model study confirmed that the expression of AR was increased and the expression of HOXA10 was reduced in the endometrium, and the HOXA10 expression was AR dependent (34). Moreover, studies have shown that the expression of AR is reduced in endometrial epithelial and stromal cells 3 months after metformin treatment in women with PCOS. In contrast, HOXA10 expression in the stromal cells treated with metformin treatment was higher than that before treatment (35).

This study has several limitations. First, testosterone level was detected to assess biochemical HA in this study, but the free androgen index was not calculated. Therefore, the total testosterone level and sex hormone-binding globulin (SHBG) will be detected to calculate the free androgen index in future research. Second, this was a single-center study. The clinical manifestations of women with PCOS are disparate in different countries, races, regions, and socioeconomic background, which need to be studied in the future. Third, the diagnostic criteria for PCOM in this study were the old criteria of Rotterdam criteria. With the publication of international evidence-based guideline for PCOS in 2023, we will design and implement studies according to the diagnostic criteria recommended by the new guidelines in the future.

## Conclusion

The clinical features of the phenotypic differences between AM-HA and AM-PCOM groups were analyzed in this study. PCOS patients with the AM-HA phenotype were vulnerable to ovulation disorders and impaired endometrial receptivity, which resulted in reduced pregnancy rate. The clinician should develop personalized treatment approaches for PCOS women with different androgen phenotypes. Treatment for HA is likely to become an effective approach for improving endometrial receptivity and fecundity disorders in patients with PCOS, which needs to be addressed clinically.

## Data Availability

The original contributions presented in the study are included in the article/supplementary material. Further inquiries can be directed to the corresponding author.
